# Fatigue, quality of life and associations with adherence to the World Cancer Research Fund guidelines for health behaviours in 5835 adults living with and beyond breast, prostate and colorectal cancer in England: A cross‐sectional study

**DOI:** 10.1002/cam4.5899

**Published:** 2023-04-06

**Authors:** Fiona Kennedy, Phillippa Lally, Natalie Ella Miller, Rana E. Conway, Anna Roberts, Helen Croker, Abigail Fisher, Rebecca J. Beeken

**Affiliations:** ^1^ Leeds Institute of Health Sciences, University of Leeds Clarendon Way Leeds LS2 9NL UK; ^2^ Department of Psychology University of Surrey Guildford Surrey GU2 7XH UK; ^3^ Behavioural Science and Health University College London Gower Street London WC1E 6BT UK; ^4^ World Cancer Research Fund International 140 Pentonville Road London N1 9FW UK

**Keywords:** cancer, diet, fatigue, health behaviours, physical activity, quality of life, survivorship

## Abstract

**Background:**

Many individuals living with and beyond cancer (LWBC) have ongoing quality of life (QoL) issues, including fatigue. The World Cancer Research Fund (WCRF) provides health behaviour recommendations for people LWBC, and there is some evidence linking adherence to these with improved QoL.

**Methods:**

Adults LWBC (specifically breast, colorectal or prostate cancer) completed a survey covering health behaviours (diet, physical activity, alcohol consumption and smoking), fatigue (FACIT‐Fatigue Scale, version 4) and a broad measure of QoL (EQ‐5D‐5L descriptive scale). Participants were categorised as meeting/not meeting WCRF recommendations, using the following cut‐offs classified as meeting the guidelines: ≥150 min physical activity/week, fruit and vegetables (≥5 portions/day), fibre (≥30 g fibre per day), free sugar (<5% of total calories from free sugar), fat (<33% total energy), red meat (<500 g/week), processed meat (none), alcohol consumption (<14 units/week) and not a current smoker. Logistic regression analyses explored associations between WCRF adherence and fatigue and QoL issues, controlling for demographic and clinical variables.

**Results:**

Among 5835 individuals LWBC (mean age: 67 years, 56% female, 90% white, breast 48%, prostate 32% and colorectal 21%), 22% had severe fatigue and 72% had 1+ issue/s on the EQ‐5D‐5L. Adhering to physical activity recommendations (odds ratio [OR] = 0.88, confidence interval [CI] = 0.77–0.99), meeting various dietary recommendations (fruit and vegetables OR = 0.79; CI = 0.68–0.91, free sugar OR = 0.85; CI = 0.76–0.96, fat OR = 0.71; CI = 0.62–0.82, red meat OR = 0.65; CI = 0.50–0.85) and not smoking (OR = 0.53, CI = 0.41–0.67) were associated with decreased odds of experiencing severe fatigue. Adhering to physical activity guidelines (OR = 0.71, CI = 0.62–0.82) was also associated with decreased odds of having 1+ QoL issue/s.

**Conclusions:**

Adherence to various WCRF recommendations, particularly the recommendation for physical activity, was associated with less fatigue and better QoL in a large UK cohort of people living with and beyond breast, colorectal or prostate cancer. Multi‐component interventions designed to support people LWBC to improve health behaviours, in line with the levels recommended by the WCRF, may also improve QoL.

## 1 INTRODUCTION

The number of individuals living with and beyond cancer (LWBC) is increasing globally due to aging populations, improved screening availability and higher precision treatments.^1^ In the United Kingdom, the number of individuals LWBC is expected to reach 4 million by 2030.^2^ Following treatment, individuals LWBC often report high levels of fatigue and decreased quality of life (QoL). Fatigue is a debilitating long‐term effect of cancer and its treatment, with profound impacts on daily lives.^3^ The rates of fatigue among those LWBC are significant, with some reports indicating that 21%–52% of individuals still experience severe fatigue up to 3 years post‐diagnosis.^4^ A study of 6952 people living with and beyond breast, prostate or colorectal cancer suggested that even up to 5–15 years post‐diagnosis individuals have deficits in various aspects of QoL and symptoms, such as fatigue and insomnia, compared to an age–sex‐matched population.^5^

Over the last 20 years, various research studies have found a relationship between meeting modifiable health behaviour recommendations (such as exercise, 5‐a‐day fruit and vegetable intake and not smoking) and higher QoL in those LWBC,^6^, ^7^ including breast, prostate and colorectal cancer. There is accumulating evidence suggesting specific benefits of physical activity on QoL and related aspects such as neuropathy and fatigue.^8^, ^9^, ^10^ Multiple systematic reviews have illustrated the beneficial impact of physical activity on QoL^11^ and fatigue,^12^ and meta‐analyses have demonstrated evidence across various cancers.^13^, ^14^ In addition, post‐diagnosis physical activity has been emphasised as having a greater impact on cancer outcomes, compared to pre‐diagnosis levels.^15^ In terms of dietary evidence, more studies have focused on fruit and vegetable intake than other aspects of diet.^16^, ^17^

In 2018, the World Cancer Research Fund (WCRF) advised that people LWBC should follow their health behaviour recommendations, which were originally designed to prevent and reduce the risk of cancer.^18^, ^19^ These recommendations centre on having a healthy diet (not only increasing fruit and vegetables, but also fibre intake; and limiting sugar, fat, red meat and processed meat intake), being physically active, maintaining a healthy body weight, only drinking alcohol in moderation, based on research which suggests that engaging in these healthy behaviours could improve long‐term cancer survival and prognosis.^8^, ^20^ Although not smoking is not one of the main WCRF recommendations, this is because it is widely acknowledged as an important predictor of cancer outcomes,^21^ including QoL,^22^ and therefore important to be included in any analysis. With the introduction of these recommendations, there has been a growing interest on the wider benefits of meeting the specific targets outlined by the WCRF. Findings from a cross‐sectional research study in the Netherlands with 150 individuals who were 2–10 years after a colorectal cancer diagnosis concluded that adherence to dietary WCRF recommendations, in particular vegetable intake, is associated with better QoL and less fatigue.^23^ Furthermore, a larger cross‐sectional registry study with 1096 individuals treated for colorectal cancer illustrated that overall WCRF adherence was associated with better global health and fatigue, and that physical activity was particularly influential.^24^ This finding was also supported in a study with 2193 mixed (breast, colorectal and gynaecological) older female cancer survivors, identified through the Iowa Women's Health Study, who similarly found those who met a greater number of WCRF recommendations had higher QoL, and physical activity illustrated the largest association.^25^ Some studies have evaluated the association using an overall WCRF adherence composite score,^26^, ^27^ whereas others have focused on specific health behaviours (e.g. physical activity,^4^, ^28^ fruit and vegetables specifically^16^). Only a few studies have looked at the associations between meeting this overall set of recommendations and QoL outcomes, and where done this has predominantly focused on those diagnosed and/or treated for colorectal cancer.^23^, ^24^ To the best of our knowledge, specific work exploring WCRF adherence and QoL has not been undertaken in the United Kingdom.

Therefore, the aim of our study was to explore associations between meeting the WCRF recommendations (including smoking) and both fatigue and QoL outcomes among individuals LWBC in England, United Kingdom.

## 2 METHODS

### 2.1 Design and setting

This UK‐based cross‐sectional study used items from the ‘Health and Lifestyle after Cancer’ survey, which is described in Beeken et al.^29^ The survey included sections covering demographics, clinical characteristics, smoking status, diet, physical activity, alcohol consumption and various QoL outcomes that are described further below.

### 2.2 Participants

Between February 2015 and January 2018, 10 hospital trusts in London and Essex were asked to identify patients who were 18+ years old and had been diagnosed with breast, prostate or colorectal cancer between 2012 and 2015. The survey was used as a way to recruit to a subsequent trial exploring a behaviour change intervention, and therefore, no specific survey sample size was determined but the survey recruitment ended when sufficient trial participants had been recruited. The inclusion criteria were deliberately broad to make it practical for hospital trusts to reach individuals who had completed, or were close to completing, primary curative treatment, and therefore LWBC. Some individuals who were diagnosed outside of these dates (1994–2017) returned the survey, and are included in this analysis.

### 2.3 Procedure

Ethical approval was obtained through the National Research Ethics Service Committee South Central—Oxford B (reference number 14/SC/1369). Participating hospitals posted letters of invitation, a paper survey and a link to an online version of the survey to eligible patients. Patients chose how they completed and returned the survey (postal or online). Completion of the survey was taken as informed consent.

### 2.4 Measures

#### 2.4.1 Demographic and clinical variables

Demographics were collected including age (years), ethnicity (survey presented 16 subcategories but for this analysis, this was dichotomised into white or non‐white due to small numbers in some groups), marital status (collected using five subcategories but dichotomised into married or not married), highest education/qualifications (collected by asking about educational/professional qualifications, categorised into no qualifications or secondary/high school and above, e.g. ~16 years+). The clinical variables collected included their most recent cancer type from breast, prostate or colorectal, time since main treatment (still having main treatment, <12 months, 12+ months, or on active surveillance), cancer spread assessed by the question ‘has this cancer spread to any other parts of your body?’ (yes/no/do not know), having had (for their most recent cancer [yes/no]) surgery, radiotherapy, chemotherapy or hormone treatment, and the number of co‐morbid conditions. For cancer spread, the response option ‘do not know’ was recoded as missing data. For the co‐morbidities question, participants were asked if they had any of the following list of health conditions (osteoporosis, diabetes, asthma, emotional or psychiatric illness, stroke, Parkinson's disease, Alzheimer's disease or dementia, lung disease, arthritis, angina, heart attack, heart murmur, irregular heart rhythm, any other heart trouble and another cancer) or any health problems not on the list. The total number of co‐morbid conditions the participant ticked was calculated.

#### 2.4.2 QoL variables

Fatigue was measured by the Functional Assessment of Chronic Illness Therapy (FACIT)‐Fatigue Scale (version 4),^30^ and QoL was measured by the EuroQol‐5 Dimension (EQ‐5D‐5L) descriptive index scale.^31^, ^32^ The FACIT‐Fatigue is a 13‐item scale developed alongside the cancer‐specific QoL FACT‐G measure,^33^ which was designed to measure cancer‐related fatigue. It has demonstrated good reliability and validity, and is appropriate for use as a standalone brief measure of fatigue.^30^ The five‐level descriptive index scale of the EQ‐5D‐5L records problems on five areas—mobility, self‐care, usual activities, pain/discomfort and anxiety/depression—often summarised into a single index score ranging from −0.5 to 1 (1 = perfect health). In this sample, due to issues with homoscedasticity and normality of residuals, both these dependent variables were dichotomised. Fatigue was dichotomised into severe fatigue (scores of 0–34) versus not severe fatigue (scores of 35–52), which has been confirmed as an appropriate threshold.^34^, ^35^ EQ‐5D‐5L index scores were dichotomised using the method outlined by Downing et al.^36^ to split participants into those who had no issues (scoring 1 ‘no problems’ on all of the five items) versus one or more problem.

#### 2.4.3 WCRF health behaviour recommendations

Overall, the survey data on all health behaviours were categorised into whether participants were meeting or not meeting each of the WCRF health behaviour recommendations, including smoking. The guidelines used to operationalise these recommendations in this study were taken from the WCRF^18^ and various national UK guidelines which are outlined in detail below.

Smoking status was collected with a single item from the Health Survey for England to categorise participants as a current smoker (not meeting) or non‐smoker (meeting).^37^ Alcohol consumption was self‐reported with two items (How often do you have a drink containing alcohol? (never [0]/monthly or less [0.23]/2–4 times per month [0.69]/2–3 times per week [2.5]/4–5 times per week [4.5]/every day [7]); and how many units of alcohol do you drink on a typical day when you are drinking? (never [0]/1–2 [1.5]/3–4 [3.5]/5–6 [5.5]/7–9 [8]/10+ [10])), adapted from The AUDIT Alcohol Consumption Questions.^38^ These two scores were multiplied to estimate the total number of units consumed on an average week. National UK guidelines for alcohol consumption recommend not exceeding more than 14 units of alcohol per week,^39^ therefore, this was operationalised as meeting the alcohol recommendation and consumption over 14 units per week was considered not meeting.

Physical activity was measured in weekly minutes of moderate to vigorous physical activity (MVPA), using items from the Godin Leisure‐Time Exercise Questionnaire (GLTEQ)^40^; minutes of strenuous activity were doubled and added to minutes of moderate activity. The validity and reliability of the GLTEQ has been confirmed in previous cancer research, compared to objective measures of physical activity.^41^ The WCRF recommendations suggest that adults undertake at least 150 min of moderate to vigorous physical activity a week,^18^ therefore, undertaking 150 min or more MVPA was categorised as meeting, whereas <150 min categorised as not meeting.

The validated Dietary Instrument for Nutrition Education Food Frequency Questionnaire (DINE FFQ)^42^ was used to measure fibre and fat, with some food items updated to reflect those currently available and items amended to facilitate red and processed meat estimation.^29^ For fibre, there are 12 items, which are summed to create a fibre score, and a score of over 40 was considered to approximately represent consuming 30 g or more of, Association of Official Analytical Collaboration (AOAC) fibre,^43^ per day, and therefore meeting the WCRF fibre recommendation. For fat, there are 19 items, which are summed to create a total fat score, and a score of below 30 is seen as equivalent to consuming less than 33% total energy,^44^ and meeting the WCRF fat recommendation. For red meat, there are two items, and the response options (once a week, 1–2 times a week, 3–5 times a week and 6+ times a week) for each were converted into a weekly score, which were summed and this score multiplied by 100 (a typical meat serving) to derive the weekly number of grams of red meat. The WCRF red meat recommendation meeting cut‐off was less than 500 g/week.^18^ For processed meat, there is one item. The WCRF processed meat recommendation meeting cut‐off was 0 g per day (i.e. no processed meat).^18^ The questionnaire also asked about sugary drinks and fruit juices,^45^, ^46^ and responses are assigned a score reflecting the average number of occasions of consumption per day (never [0]/ once a week [0.14]/2–3 times a week [0.36]/4–6 times a week [0.71]/ once a day [1], twice a day [2] or 3+ times a day [3.5]). The amount of sugar in a sugary drink/fruit juice was estimated respectively (14.4 g/250 mL/12.9 g/150 mL), which was multiplied by the consumption per day value and summed together to give an estimate of grams of sugar in drinks per day. Finally, an item exploring teaspoons of added sugar to estimate free sugar was used, which was multiplied by 5, and added to the sugar in drinks to give a total grams of added sugar per day. This is estimated as around a third of an individual's sugar intake (a third of free sugar came from drinks and preserves in the National Dietary and Nutrition Survey^47^), therefore this figure is multiplied by 3 to give a total estimate of total free sugars per day. This total free sugars per day was then converted into % total calories from free sugar by multiplying the total free sugars value by 4 (as sugar has approximately 4 calories per gram), divided it respectively for women/men (out of 2000/2500 kcal), and multiplied by 100. The WCRF sugar recommendation was operationalised as being met if individuals had less than 5% calories from free sugar.

Finally, two items were added to measure the number of daily portions of fruit and vegetables,^48^ and the response options were then converted to a daily amount (less than one per week [0.07]/one per week [0.14]/2–3 per week [0.36]/4–6 per week [0.71]/one per day [1]/two per day [2]/3+ per day [3.5]). This method of scoring has been used in other similar studies^45^ and is summed to create a daily fruit and vegetable score, and five portions/day or more were considered meeting the WCRF recommendation.

### 2.5 Statistical analysis

IBM Statistical Package for the Social Sciences (SPSS) version 26 was used. Descriptive statistics were run for demographic, clinical characteristics and health behaviours.

Missing value analysis found that 5.5% of 518,348 values were missing and 27.8% of 5835 cases had at least one piece of missing data. Multiple imputation with 20 iterations was conducted to account for missing data in relation to all the variables included in the analysis.^49^ This imputation was conducted twice, with similar results, and therefore the first imputed data set was used.

Unadjusted and a priori confounder‐adjusted logistic regression models were used to assess associations between the adherence to WCRF categories (dietary, physical activity, smoking and alcohol) and the dichotomous‐dependent variables (fatigue and QoL). First, a series of unadjusted regressions were run for each of the independent variables individually and with each dependent variable, with no covariates included in each model. Then, two separate logistic regressions were run including all independent variables and covariates for each dependent variable (age, ethnicity, education/qualifications, marital status, cancer type, time since main treatment, cancer spread, surgery, radiotherapy, chemotherapy, hormone therapy and number of co‐morbidities). ‘Gender’ was not included as a covariate due to the potential for multicollinearity, as ‘cancer type’ and ‘gender’ were identical in the breast (all female) and prostate cancer samples (all male). The final logistic regressions were repeated in the completers sample (*N* = 1946 fatigue model/*N* = 1939 QoL model) to explore if similar results were achieved.

## 3 RESULTS

### 3.1 Sample characteristics

A total of 5835 surveys were returned (response rate 42.8%, out of 13,645 distributed). No data were collected about the non‐responders. Participant characteristics are presented in Table 1. Mean age was 67 years, 56% were female, 90% were white and 70% were within 1–5 years post‐completion of main treatment. Almost half had been diagnosed with breast cancer (*n* = 2786, 48%), 32% prostate (*n* = 1839) and 21% colorectal cancer (*n* = 1210).

**TABLE 1:** Demographic and clinical characteristics among the sample of breast, prostate and colorectal participants.

	Total sample
	*N* = 5835
Questionnaire format *N* (%)	
Paper questionnaire	5801 (99.4)
Online survey	34 (0.6)
Gender *N* (%)	
Male	2553 (43.8)
Female	3266 (56.0)
Missing	16 (0.3)
Age	
Mean (SD)	67.4 (11.8)
Missing (*N*)	36 (0.6)
Highest education/qualifications *N* (%)	
None/no qualifications/below school leaving age	1709 (29.3)
Secondary/High school or above	3576 (61.3)
Missing	550 (9.4)
Marital status *N* (%)	
Married	4037 (69.2)
Other (e.g. divorced, single, separated and widowed)	1781 (30.5)
Missing	17 (0.3)
Ethnicity—dichotomised *N* (%)	
White	5249 (90.0)
Non‐white	554 (9.5)
Missing	32 (0.5)
Months since most recent cancer diagnosis	
Mean (SD)	35.5 (13.6)
Cancer type *N* (%)	
Breast	2786 (47.7)
Prostate	1839 (31.5)
Colorectal	1210 (20.7)
Cancer spread *N* (%)	
Yes	558 (9.6)
No	4498 (77.1)
Do not know	373 (6.4)
Missing	406 (7.0)
Time since main treatment *N* (%)	
Still having main treatment	490 (8.4)
<12 months	495 (8.5)
1–5 years	4122 (70.6)
On active surveillance	525 (9.0)
Do not know/cannot remember	54 (0.9)
Missing	149 (2.6)
Total co‐morbidities	
Mean	1.3
None *n* (%)	1849 (31.7)
1	1991 (34.1)
2	1120 (19.2)
>3	875 (15.0)
Missing	0

The descriptive statistics for physical activity, smoking and the dietary variables are shown in Table 2. Lower adherence to WCRF recommendations were seen in relation to physical activity (30.7%), fibre (11.4%), fruit and vegetables (28.4%), processed meat (45.2%), and fat (39.5%), compared to the other recommendations (e.g. free sugar 56.5%, alcohol 83.1%, red meat 86.3% and smoking 93.3%). In total, 22% of participants were categorised as having severe fatigue (0–34) compared to 56% not having severe fatigue (22% missing), and 72% had one or more problems on the EQ‐5D‐5L descriptive scale compared to 22% having no problems (6% missing).

**TABLE 2:** Meeting of WCRF health behaviours in study participants.

WCRF recommendations^a^	Total
	*n* = 5835
Dietary variables	
Fruit and vegetables *n* (%)	
Meeting	1655 (28.4)
Not meeting	3977 (68.2)
Missing	203 (3.5)
Fibre *n* (%)	
Meeting	668 (11.4)
Not meeting	3919 (67.2)
Missing	1248 (21.4)
Free sugar *n* (%)	
Meeting	2711 (56.5)
Not meeting	2673 (45.8)
Missing	451 (7.7)
Fat *n* (%)	
Meeting	2303 (39.5)
Not meeting	1769 (30.3)
Missing	1763 (30.2)
Red meat *n* (%)	
Meeting	5035 (86.3)
Not meeting	139 (2.4)
Missing	661 (11.3)
Processed meat *n* (%)	
Meeting	2640 (45.2)
Not meeting	2861 (49.0)
Missing	334 (5.7)
Alcohol *n* (%)	
Meeting	4848 (83.1)
Not meeting	714 (12.2)
Missing	273 (4.7)
Physical activity *n* (%)	
≥150 min per week/Meeting	1790 (30.7)
<150 min per week/Not meeting	3359 (57.6)
Missing	686 (11.8)
Smoking *n* (%)	
Not smoking/Meeting	5445 (93.3)
Smoking/Not meeting	347 (5.9)
Missing	43 (0.7)

Abbreviations: MVPA, moderate to vigorous physical activity; WCRF, World Cancer Research Fund.

^a^ Meeting WCRF cut‐offs were as follows: ≥5 portions of fruit and vegetables/day, ≥30 g fibre/day, <5% of calories from free sugar, <33% energy from fat, <500 g/week red meat, 0 g/day processed meat, ≤14 units/week alcohol, not smoking, ≥150 min MVPA/week.

### 3.2 Logistic regression results

#### 3.2.1 Main analysis

In terms of experiencing fatigue, after adjusting for potential covariates, participants who were meeting recommendations for physical activity, fruit and vegetables, free sugar, fat intake and red meat had reduced odds of having severe fatigue by 12%–35% (Table 3). Furthermore, not smoking decreased the likelihood of severe fatigue by 47%. The completer analysis (Data S1) showed broadly similar results, although some of the associations were no longer statistically significant.

**TABLE 3:** Logistic regression analysis for fatigue among study participants (*n* = 5835).

	Unadjusted			Adjusted^a^		
	OR	CI	*p*	OR	CI	*p*
Physical activity						
Not meeting	1.00			1.00	‐	‐
Meeting	0.81	0.72, 0.92	<0.01*	0.88	0.77, 0.99	0.04*
Fruit and vegetables						
Not meeting	1.00			1.00	‐	‐
Meeting	0.74	0.65, 0.84	<0.01*	0.79	0.68, 0.91	<0.001*
Fibre						
Not meeting	1.00			1.00	‐	‐
Meeting	1.22	1.04, 1.42	0.01*	1.15	0.97, 1.36	0.12
Free sugar						
Not meeting	1.00			1.00	‐	‐
Meeting	0.77	0.69, 0.86	<0.01*	0.85	0.76, 0.96	0.01*
Fat						
Not meeting	1.00			1.00	‐	‐
Meeting	0.66	0.58, 0.74	<0.01*	0.71	0.62, 0.82	<0.001*
Red meat						
Not meeting	1.00			1.00	‐	‐
Meeting	0.52	0.41, 0.66	<0.01*	0.65	0.50, 0.85	0.002*
Processed meat						
Not meeting	1.00			1.00	‐	‐
Meeting	0.97	0.86, 1.10	0.66	1.09	0.95, 1.25	0.22
Alcohol						
Not meeting	1.00			1.00	‐	‐
Meeting	1.24	1.03, 1.48	0.02*	1.01	0.83, 1.23	0.92
Smoking						
Not meeting	1.00			1.00	‐	‐
Meeting	0.48	0.39, 0.60	<0.01*	0.53	0.41, 0.67	<0.001*

*Note*: Severe fatigue as target group, 1 = severe fatigue 0–34; 0 = not severe fatigue 35–52.

Abbreviations: CI, confidence interval; OR, odds ratio.

^a^ Adjusted for age (years), ethnicity (white, non‐white), education (none/no qualifications, secondary/high school or above), marital status (married, other), cancer type (breast, prostate and colorectal), time since main treatment (still having treatment, <12 months, 12+ months and active surveillance), cancer spread (spread, no spread), surgery (yes, no), radiotherapy (yes, no), chemotherapy (yes, no), hormone therapy (yes, no) and number of co‐morbidities (total).

* *p* < 0.05.

In terms of experiencing QoL issues, after adjusting for potential covariates, participants who were meeting the recommendations for physical activity, fat intake and smoking had lower odds of having one or more QoL issues by 29%, 18% and 33% respectively (Table 4). The completer analysis (Data S1) showed similar results, although the association of smoking and fat was no longer statistically significant, and meeting the free sugar recommendation was associated with decreased odds of QoL issues.

**TABLE 4:** Logistic regression analysis for EQ‐5D‐5L problems among study participants (*n* = 5835).

	Unadjusted			Adjusted^a^		
	OR	CI	*p*	OR	CI	*p*
Physical activity						
Not meeting	1.00			1.00	‐	‐
Meeting	0.65	0.56, 0.74	<0.01*	0.71	0.62, 0.82	<0.001*
Fruit and vegetables						
Not meeting	1.00			1.00	‐	‐
Meeting	0.89	0.77, 1.02	0.08	0.89	0.77, 1.04	0.14
Fibre						
Not meeting	1.00			1.00	‐	‐
Meeting	1.19	0.99, 1.42	0.06	1.16	0.95, 1.41	0.14
Free sugar						
Not meeting	1.00			1.00	‐	‐
Meeting	0.86	0.76, 0.98	0.02*	0.89	0.77, 1.03	0.11
Fat						
Not meeting	1.00			1.00	‐	‐
Meeting	0.77	0.68, 0.88	<0.01*	0.82	0.70. 0.96	0.01*
Red meat						
Not meeting	1.00			1.00	‐	‐
Meeting	0.87	0.66, 1.16	0.35	0.98	0.72, 1.35	0.91
Processed meat						
Not meeting	1.00			1.00	‐	‐
Meeting	0.89	0.79, 1.01	0.08	0.94	0.81, 1.09	0.40
Alcohol						
Not meeting	1.00			1.00	‐	‐
Meeting	1.04	0.86, 1.25	0.68	0.86	0.70, 1.05	0.14
Smoking						
Not meeting	1.00			1.00	‐	‐
Meeting	0.63	0.47, 0.85	0.01*	0.67	0.49, 0.92	0.01*

*Note*: EQ‐5D‐5L issues as target group, 1 = 1 or more issues; 0 = no issues.

Abbreviations: CI, confidence interval; OR, odds ratio.

^a^ Adjusted for age (years), ethnicity (white, non‐white), education (none/no qualifications, secondary/high school or above), marital status (married, other), cancer type (breast, prostate and colorectal), time since main treatment (still having treatment, <12 months, 12+ months and active surveillance), cancer spread (spread, no spread), surgery (yes, no), radiotherapy (yes, no), chemotherapy (yes, no), hormone therapy (yes, no) and number of co‐morbidities (total).

* *p* < 0.05.

## 4 DISCUSSION

To the best of our knowledge, this is one of the few large‐scale studies illustrating the relationship between adherence to WCRF recommendations and fatigue and QoL outcomes among individuals living with and beyond breast, prostate and colorectal cancer, and one of the first studies focused on this within the United Kingdom. Adherence to these recommendations, and particularly adherence to the recommendations to be physically active and not smoke, was associated with both higher QoL and lower levels of fatigue.

Previous studies have often been within a single cancer (e.g. colorectal^23^), mixed sample populations with small samples^6^ and not UK‐based. For example, in 2004, Blanchard et al.^6^ reported a study that determined that meeting the WCRF physical activity recommendation was associated with higher QoL among 316 breast, colorectal and prostate patients. However, Blanchard's study was undertaken in the United States (which has private health care services) and among individuals diagnosed with cancer between the years 1990–1998. This study was among UK individuals diagnosed more recently and therefore the results of this study make an important contribution to the growing evidence illustrating the importance of focusing on modifiable health behaviours among individuals LWBC.

Only 22% of participants met the threshold for severe fatigue, which is lower than the 51.5% reported in a previous study,^4^ although the studies differed in terms of the measure of fatigue used (EORTC fatigue vs. FACIT‐F subscale) and time since diagnosis. Matias et al. included breast, prostate and colorectal patients who were specifically 2 years post‐diagnosis, whereas individuals in this study were on average further post‐diagnosis (mean 3 years). Almost three quarters of this sample had ongoing QoL problems as measured by the EQ‐5D‐5L, which is higher than the 65.5% proportion reporting problems in a large UK study by Downing et al.^36^ The consistent relationship between physical activity and both outcomes (fatigue, QoL) in this study strengthens the growing impetus for the development of interventions to specifically target this as suggested in earlier WCRF research.^24^, ^25^ Physical activity is known to have many benefits among those LWBC, and therefore further work should be undertaken to support patients to build and maintain good physical activity habits post‐treatment. However, this study also highlights the need to consider various health behaviours because in addition to physical activity, smoking was related to both outcomes and various aspects of diet (e.g. free sugar, fat, fruit and vegetable) were also associated. This contrasts with previous diet‐focused research, which has often only emphasised the importance of fruit and vegetable consumption.^16^, ^23^

This study also reinforces the importance of not just focusing on a composite score, which has previously been highlighted as disregarding the contribution that individual modifiable health behaviours may have,^27^ and therefore future work should compare individual health behaviours with composite scores in terms of effects on QoL outcomes. However, it is also important to highlight that health behaviours are likely to cluster and have synergistic effects,^50^, ^51^ and therefore similar to previous calls in colorectal cancer,^27^ we would suggest that multi‐component interventions targeting multiple health behaviours is a fruitful direction to explore among individuals LWBC.^29^

The findings of this research also highlight the importance of emphasising the message around health behaviours to both individuals LWBC and also their primary and secondary healthcare professionals. Previous research has highlighted that individuals LWBC do not often receive lifestyle advice, despite a desire to receive it,^52^ and health professionals have raised concerns and identified barriers to the provision of behavioural advice in clinical practice.^53^ Research is ongoing to evaluate multi‐component habit‐based behavioural interventions and physical activity interventions among individuals LWBC in the United Kingdom^29^, ^54^ and elsewhere,^55^ which this study would support.

Limitations of this study include the relatively high level of missing data, especially for the fatigue variable (22% missing) and some WCRF variables (e.g. fibre 20%, fat 30% and physical activity over 10% missing). The data were imputed to reduce possible bias,^49^ and the results of the sensitivity analysis among completers illustrate some subtle differences, which is likely due to reduced power. There was also only a small proportion of ethnicities other than white (9.5%) in our sample, and therefore, it is uncertain whether the results are generalisable to all ethnic groups. Overall, the response rate was 42.8%, and although this is comparable to other studies,^16^ it is lower than the 63% response rate in a large colorectal UK survey.^36^ Furthermore, it was not possible to capture any information about patients who declined because the data collection process was organised by hospitals, which limits the generalisability. Reliance on individuals self‐reported health behaviours may mean both underestimation (e.g. alcohol consumption) and overestimation (e.g. physical activity) is possible, and future work should aim to use more objective measures where possible (e.g. accelerometers). The use of EQ‐5D‐5L descriptive scale to capture QoL was a generic, rather than a cancer‐specific measure, which may not have identified all aspects of QoL relevant for individuals LWBC. Finally, the study was cross‐sectional, which means that causal inferences cannot be made, and the relationship between health behaviours (e.g. physical activity) and fatigue is likely to be bidirectional.^56^ Much of the previous research in this area has been cross‐sectional in nature, apart from a recent series of articles by Kenkhuis et al. exploring longitudinal relationships among colorectal cancer survivors' post‐treatment adherence with WCRF and QoL,^27^, ^28^, ^57^ which suggests that higher MVPA was longitudinally associated with higher QoL.^28^ Furthermore, Eyl‐Armbruster et al. found that positive changes in lifestyle behaviours (composite score) among colorectal cancer survivors' from baseline to 5 years was associated with better functioning and lower symptom burden at 5 years follow‐up.^58^ Future prospective studies should seek to better understand the direction of these relationships in different groups of cancer survivors, and intervention studies should aim to explore whether adhering to these recommendations results in better patient QoL outcomes in the longer term.

## 5 CONCLUSIONS

Despite the limitations, this study represents one of the first and largest UK‐based cross‐sectional surveys exploring associations between WCRF health behaviours and QoL outcomes among a mixed group of individuals diagnosed with breast, prostate and colorectal cancer. The results illustrate the importance of focusing on modifiable health behaviours in those LWBC, and the development and exploration of multi‐component interventions to support those LWBC to live well after their diagnosis and treatment.

## AUTHOR CONTRIBUTIONS

**Fiona Kennedy:** Formal analysis (lead); writing – original draft (lead); writing – review and editing (lead). **Phillippa Lally:** Data curation (lead); formal analysis (supporting); supervision (lead); writing – review and editing (equal). **Natalie Ella Miller:** Formal analysis (supporting); writing – review and editing (equal). **Rana E. Conway:** Formal analysis (equal); writing – review and editing (equal). **Anna Roberts:** Formal analysis (equal); writing – review and editing (equal). **Helen Croker:** Conceptualization (equal); formal analysis (equal); methodology (equal); writing – review and editing (equal). **Abigail Fisher:** Conceptualization (equal); formal analysis (equal); funding acquisition (equal); methodology (equal); supervision (equal); writing – review and editing (equal). **Rebecca J. Beeken:** Conceptualization (equal); formal analysis (equal); funding acquisition (equal); methodology (equal); supervision (equal); writing – review and editing (equal).

## FUNDING INFORMATION

This work was funded by Cancer Research UK (grant number C43975/A27498). The charity had no influence on the data collection, interpretation or reporting.

## CONFLICT OF INTEREST STATEMENT

Helen Croker reports funding from the World Cancer Research Fund. Rebecca J. Beeken reports funding from Yorkshire Cancer Research. The other authors have no conflicts of interest to declare that are relevant to the content of this article.

## ETHICS APPROVAL STATEMENT

Ethical approval was obtained through the National Research Ethics Service Committee South Central—Oxford B (reference number 14/SC/1369).

## PATIENT CONSENT STATEMENT

The following statement was provided on the questionnaire: ‘By completing this questionnaire you are consenting to your anonymous information being used for research on lifestyle in people diagnosed with cancer’.

## Supporting information


Data S1.

## Data Availability

The data sets generated during and/or analysed during this study are available from the corresponding author on reasonable request.
