# Supplementary material for: Fatigue, quality of life and associations with adherence to the World Cancer Research Fund guidelines for health behaviours in 5835 adults living with and beyond breast, prostate and colorectal cancer in England: A cross‐sectional study
Source: Cancer Med. 2023 Apr 6;12(11):12705–16. doi: 10.1002/cam4.5899 (PMC10278485; doi:10.1002/cam4.5899)
Supplement: Supplementary file 1 — Data S1. [file CAM4-12-12705-s001.docx]

**Additional File 1**

*Additional File 1: Table 1 – Logistic regression analyses for participants with complete data on all variables – Fatigue*^#^ *analysis (n=1946)*

| WRCF categories  (1=meets, 0=not) | Unadjusted | | | Adjusted † | | |
| --- | --- | --- | --- | --- | --- | --- |
|  | *OR* | *CI* | *p* | *OR* | *CI* | *p* |
| Physical activity | 0.44 | 0.35, 0.54 | <0.001* | 0.47 | 0.36, 0.60 | <0.001* |
| Fibre | 0.95 | 0.70, 1.29 | 0.76 | 1.02 | 0.73, 1.42 | 0.93 |
| Fruit & veg | 0.88 | 0.70, 1.09 | 0.24 | 0.97 | 0.76, 1.24 | 0.78 |
| Red meat | 0.89 | 0.45, 1.75 | 0.74 | 1.29 | 0.61, 2.72 | 0.51 |
| Processed meat | 1.03 | 0.84, 1.26 | 0.77 | 1.05 | 0.83, 1.33 | 0.68 |
| Free Sugar | 0.81 | 0.66, 0.99 | 0.04* | 0.90 | 0.72, 1.12 | 0.33 |
| Fat | 0.83 | 0.68, 1.02 | 0.07 | 0.80 | 0.63, 1.02 | 0.08 |
| Alcohol | 1.21 | 0.89, 1.63 | 0.23 | 0.96 | 0.69, 1.33 | 0.80 |
| Smoking | 0.44 | 0.29, 0.66 | <0.001* | 0.48 | 0.31, 0.76 | 0.002* |

** p<0.05*

*^#^ Severe fatigue as target group, 1 = severe fatigue 0-34; 0 = not severe fatigue 35-52*

† Adjusted for age (years), ethnicity (white, non-white), education (none/no qualifications, secondary/high school or above), marital status (married, other), cancer type (breast, prostate, colorectal), time since main treatment (still having treatment, <12 months, 12+months, active surveillance), cancer spread (spread, no spread), surgery (yes, no), radiotherapy (yes, no), chemotherapy (yes, no), hormone therapy (yes, no), number of co-morbidities (total).

*Additional File 1: Table 2 – Logistic regression analyses for participants with complete data on all variables – EQ5D^#^ analysis (n=1939)*

| WRCF categories  (1=meets, 0=not) | Unadjusted | | | Adjusted† | | |
| --- | --- | --- | --- | --- | --- | --- |
|  | *OR* | *CI* | *P* | *OR* | *CI* | *P* |
| Physical activity | 0.45 | 0.37, 0.55 | <.001* | 0.54 | 0.43, 0.68 | <0.001* |
| Fibre | 1.19 | 0.87, 1.61 | 0.28 | 1.17 | 0.83, 1.65 | 0.37 |
| Fruit & veg | 0.88 | 0.71, 1.10 | 0.26 | 0.94 | 0.74, 1.20 | 0.64 |
| Red meat | 0.75 | 0.36, 1.57 | 0.44 | 0.86 | 0.38, 1.94 | 0.72 |
| Processed meat | 0.90 | 0.73, 1.10 | 0.30 | 0.94 | 0.75, 1.19 | 0.63 |
| Free Sugar | 0.73 | 0.60, 0.90 | 0.003* | 0.80 | 0.64, 1.00 | 0.05* |
| Fat | 0.81 | 0.66, 0.99 | 0.04* | 0.90 | 0.71, 1.15 | 0.40 |
| Alcohol | 1.10 | 0.82, 1.46 | 0.53 | 0.68 | 0.68, 1.28 | 0.66 |
| Smoking | 0.53 | 0.31, 0.92 | 0.02* | 0.32 | 0.32, 1.02 | 0.06 |

** p<0.05*

*^#^ EQ5D issues as target group, 1 = 1 or more issues; 0 = no issues*

† Adjusted for age (years), ethnicity (white, non-white), education (none/no qualifications, secondary/high school or above), marital status (married, other), cancer type (breast, prostate, colorectal), time since main treatment (still having treatment, <12 months, 12+months, active surveillance), cancer spread (spread, no spread), surgery (yes, no), radiotherapy (yes, no), chemotherapy (yes, no), hormone therapy (yes, no), number of co-morbidities (total).
